# Detection of high CD44 expression in oral cancers using the novel monoclonal antibody, C_44_Mab-5

**DOI:** 10.1016/j.bbrep.2018.03.007

**Published:** 2018-04-12

**Authors:** Shinji Yamada, Shunsuke Itai, Takuro Nakamura, Miyuki Yanaka, Mika K. Kaneko, Yukinari Kato

**Affiliations:** a Department of Antibody Drug Development, Tohoku University Graduate School of Medicine, 2-1 Seiryo-machi, Aoba-ku, Sendai, Miyagi 980-8575, Japan; b New Industry Creation Hatchery Center, Tohoku University, 2-1, Seiryo-machi, Aoba-ku, Sendai, Miyagi 980-8575, Japan

**Keywords:** mAb, monoclonal antibody, CBIS, cell-based immunization and screening, SCC, squamous cell carcinoma, ACC, adenoid cystic carcinoma, MEC, mucoepidermoid carcinoma, DMEM, Dulbecco's Modified Eagle's Medium, EDTA, ethylenediaminetetraacetic acid, BSA, bovine serum albumin, PBS, phosphate-buffered saline, FBS, fetal bovine serum, DAB, 3,3-diaminobenzidine tetrahydrochloride, CD44, Monoclonal antibody, Immunohistochemistry, Oral cancer

## Abstract

CD44 is a transmembrane glycoprotein that regulates a variety of genes related to cell-adhesion, migration, proliferation, differentiation, and survival. A large number of alternative splicing isoforms of CD44, containing various combinations of alternative exons, have been reported. CD44 standard (CD44s), which lacks variant exons, is widely expressed on the surface of most tissues and all hematopoietic cells. In contrast, CD44 variant isoforms show tissue-specific expression patterns and have been extensively studied as both prognostic markers and therapeutic targets in cancer and other diseases. In this study, we immunized mice with CHO-K1 cell lines overexpressing CD44v3-10 to obtain novel anti-CD44 mAbs. One of the clones, C_44_Mab-5 (IgG_1_, kappa), recognized both CD44s and CD44v3-10. C_44_Mab-5 also reacted with oral cancer cells such as Ca9-22, HO-1-u-1, SAS, HSC-2, HSC-3, and HSC-4 using flow cytometry. Moreover, immunohistochemical analysis revealed that C_44_Mab-5 detected 166/182 (91.2%) of oral cancers. These results suggest that the C_44_Mab-5 antibody may be useful for investigating the expression and function of CD44 in various cancers.

## Highlights

• CD44 is related to migration, proliferation, and differentiation of cancer cells.
• Sensitive and specific C_44_Mab-5 mAb against CD44s was produced.
• C_44_Mab-5 mAb reacted with oral cancer cells sensitively in flow cytometry.
• C_44_Mab-5 mAb detected 166/182 (91.2%) of oral cancers in Immunohistochemistry.

## 1 Introduction

CD44 is a transmembrane glycoprotein expressed on many cell types, including endothelial cells, epithelial cells, fibroblasts, keratinocytes, and leukocytes [1]. CD44 is believed to play important roles in physiological processes such as cell proliferation, adhesion, migration, and lymphocyte activation [2]. The CD44 gene consists of 20 exons [3], with ten constitutively expressed to produce the smallest isoform, which is the standard form of CD44 (CD44s). The other possible isoforms are categorized as CD44 variants (CD44v) and are generated by alternatively spliced transcripts [4]. The diversity of the CD44 protein is also augmented by post-translational modifications such as *N*- and *O*-glycosylation and heparan sulfate [5], [6].

Cancer cells often express a large variety of CD44 variants, particularly in the advanced stages. Indeed, the CD44v6 isoform was first identified as one of the metastatic determinants in cancer. CD44v6-specific monoclonal antibodies (mAbs) have been shown to inhibit the metastasis of rat pancreatic tumor cells [7], [8]. Furthermore, the transfection of CD44v4-7 cDNA has been shown to confer a metastatic phenotype to non-metastatic cells [9]. In addition, CD44v6 isoforms have been reported to act as co-receptors for receptor tyrosine kinases (RTKs), such as Met and VEGFR-2 [10], [11], [12]. Another variant isoform, CD44v3, is able to bind several heparan sulfate-binding growth factors, such as FGFs and HB-EGF [13], [14]. These studies suggest that the co-receptor function of CD44v for RTKs is required for tumor progression. Several reports have also indicated that CD44v likely functions as a prognostic marker in many tumors, including lung, colorectal, breast, hepatocellular, and head and neck cancers [15], [16], [17], [18], [19]. Considering that CD44 has multifunctional roles and a promising prognostic value in various cancers, targeting CD44 for cancer therapy may prove to be a promising approach. MAbs, which neutralize the binding of hyaluronic acid to CD44, have been shown to inhibit anchorage-independent growth of murine mammary carcinoma cells and human colon carcinoma cells, and to induce apoptosis *in vitro* [20]. In addition, mAbs against CD44 or CD44v have been demonstrated to exert significant antitumor activity in *in vivo* animal models of human xenograft tumors [21], [22]. Although a number of studies have been conducted on CD44, CD44v isoforms have not been fully characterized. This is, in part, due to the lack of critical probes needed for the specific detection of CD44v isoforms, as limited antibodies against CD44 variant exons are commercially available. Therefore, sensitive antibodies to CD44v-specific isoforms are necessary.

Recently, we immunized mice with cat podoplanin-expressed CHO-K1 cells (CHO/catPDPN) and performed screening using CHO/catPDPN in flow cytometry [23]. This method was named as the Cell-Based Immunization and Screening (CBIS) method. Using this CBIS method, we obtained highly sensitive mAbs against various membrane proteins. In this study, we aimed to develop a novel anti-CD44 mAb using the CBIS method.

## 2 Materials and methods

### 2.1 Cell lines and plasmids

Ca9-22, HO-1-u-1, SAS, HSC-2, HSC-3, and HSC-4 cells were obtained from the Japanese Collection of Research Bioresources Cell Bank (Osaka, Japan). CHO-K1, LN229, and P3U1 cell lines were obtained from the American Type Culture Collection (ATCC, Manassas, VA).

CD44v3-10 open reading frame (ORF) was provided by the RIKEN BRC through the National Bio-Resource Project of the MEXT, Japan. CD44s ORF was amplified from LN229 cDNA using HotStar HiFidelity Polymerase Kit (Qiagen Inc., Hilden, Germany). CD44v3-10 ORF and CD44s ORF were subcloned into pCAG-cRAP-MAP vector possessing C-terminal RAP/MAP tag and pCAG-ssPA16 vector possessing signal sequence and N-terminal PA16 tag (GLEGGVAMPGAEDDVV), respectively. These plasmids were named as pCAG-CD44v3-10 and pCAG-ssPA16-CD44s, respectively, and were transfected into CHO-K1 cells using a Neon transfection system (Thermo Fisher Scientific, Inc., Waltham, MA). The stable transfectant of CHO/CD44v3-10 was established by a cell sorter (SH800; Sony Corp., Tokyo, Japan).

Ca9-22, HSC-2, HSC-3, and HSC-4 cells were cultured in Dulbecco's Modified Eagle's Medium (DMEM; Nacalai Tesque, Kyoto, Japan), and HO-1-u-1, SAS, CHO-K1, CHO/CD44v3-10, CHO/CD44s, and P3U1 cell lines were cultured in RPMI 1640 medium (Nacalai Tesque, Inc.) at 37 °C in a humidified atmosphere containing 5% CO_2_ and 95% air, both of which were supplemented with 10% heat-inactivated fetal bovine serum (FBS; Thermo Fisher Scientific, Inc.). One hundred units/mL penicillin, 100 μg/mL streptomycin, and 25 μg/mL amphotericin B (Nacalai Tesque, Inc.) were added to the culture medium. G418 (0.5 mg/mL; FUJIFILM Wako Pure Chemical Industries Ltd., Osaka, Japan) was added to the culture medium of CHO/CD44v3-10 and CHO/CD44s.

### 2.2 Animals and human tissues

Female 4-week-old BALB/c mice were purchased from CLEA Japan (Tokyo, Japan) and kept under specific pathogen-free (SPF) conditions. The Animal Care and Use Committee of Tohoku University approved all animal experiments described in this study. Oral cancer tissue arrays were purchased from US Biomax, Inc. (Rockville, MD): Cases 1–38 from Cat. # OR480, Cases 39–85 from Cat. # OR601b or from Cybrdi, Inc. (Frederick, MD): Cases 86–156 from Cat. # 27-10-001. The study examined 26 patients (Case-157-182) with oral cancer who underwent surgery at the Tokyo Medical and Dental University. The Tokyo Medical and Dental University Institutional Review Board reviewed and approved the use of human cancer tissues, and written informed consent was obtained for using the human cancer tissue samples.

### 2.3 Hybridoma production

Two BALB/c mice were immunized using intraperitoneal (i.p.) injections of CHO/CD44v3-10 (1 × 10^8^ cells) together with Imject Alum (Thermo Fisher Scientific Inc.). After three additional immunizations, a booster injection of CHO/CD44v3-10 was intraperitoneally administered 2 days before harvesting the spleen cells. Spleen cells were then fused with P3U1 cells using PEG1500 (Roche Diagnostics, Indianapolis, IN). The resulting hybridomas were grown in RPMI medium supplemented with 10% FBS and hypoxanthine, aminopterin, thymidine selection medium supplement (Thermo Fisher Scientific Inc.), and 5% BriClone Hybridoma Cloning Medium (QED Bioscience Inc., San Diego, CA). One hundred units/mL penicillin, 100 μg/mL streptomycin, and 25 μg/mL amphotericin B (Nacalai Tesque, Inc.) were added to the medium. Plasmocin (5 μg/mL; InvivoGen, San Diego, CA) was also used to prevent *Mycoplasma* contamination. Culture supernatants were screened by SA3800 Cell Analyzers (Sony Corp.) using CHO-K1 and CHO/CD44v3-10. MAbs were purified from the supernatants of hybridomas and cultured in Hybridoma-SFM medium (Thermo Fisher Scientific Inc.) using Protein G Sepharose 4 Fast Flow (GE Healthcare UK Ltd, Buckinghamshire, England).

### 2.4 Flow cytometry

Cells were harvested by briefly exposing to 0.25% trypsin/1-mM ethylenediaminetetraacetic acid (EDTA; Nacalai Tesque, Inc.). After washing with 0.1% bovine serum albumin (BSA)/phosphate-buffered saline, the cells were treated with 10 μg/mL of primary antibodies, for 30 min at 4 °C and subsequently with Alexa Fluor 488-conjugated anti-mouse IgG (1:1000; Cell Signaling Technology, Inc., Danvers, MA). Fluorescence data were collected using EC800 Cell Analyzers (Sony Corp.).

### 2.5 Immunohistochemical analyses

Histological sections of 4-μm thickness were autoclaved in citrate buffer (pH 6.0; Nichirei Biosciences, Inc., Tokyo, Japan) for 20 min. Sections were then incubated with 1 μg/mL of primary mAbs for 1 h at room temperature, treated using an Envision+ kit (Agilent Technologies Inc.) for 30 min. Color was developed using 3,3-diaminobenzidine tetrahydrochloride (DAB; Agilent Technologies Inc.) for 2 min, and counterstained with hematoxylin (FUJIFILM Wako Pure Chemical Industries Ltd.).

## 3 Results and discussion

### 3.1 Production of novel anti-CD44 mAbs using the Cell-Based Immunization and Screening (CBIS) method

**Fig. 1:**
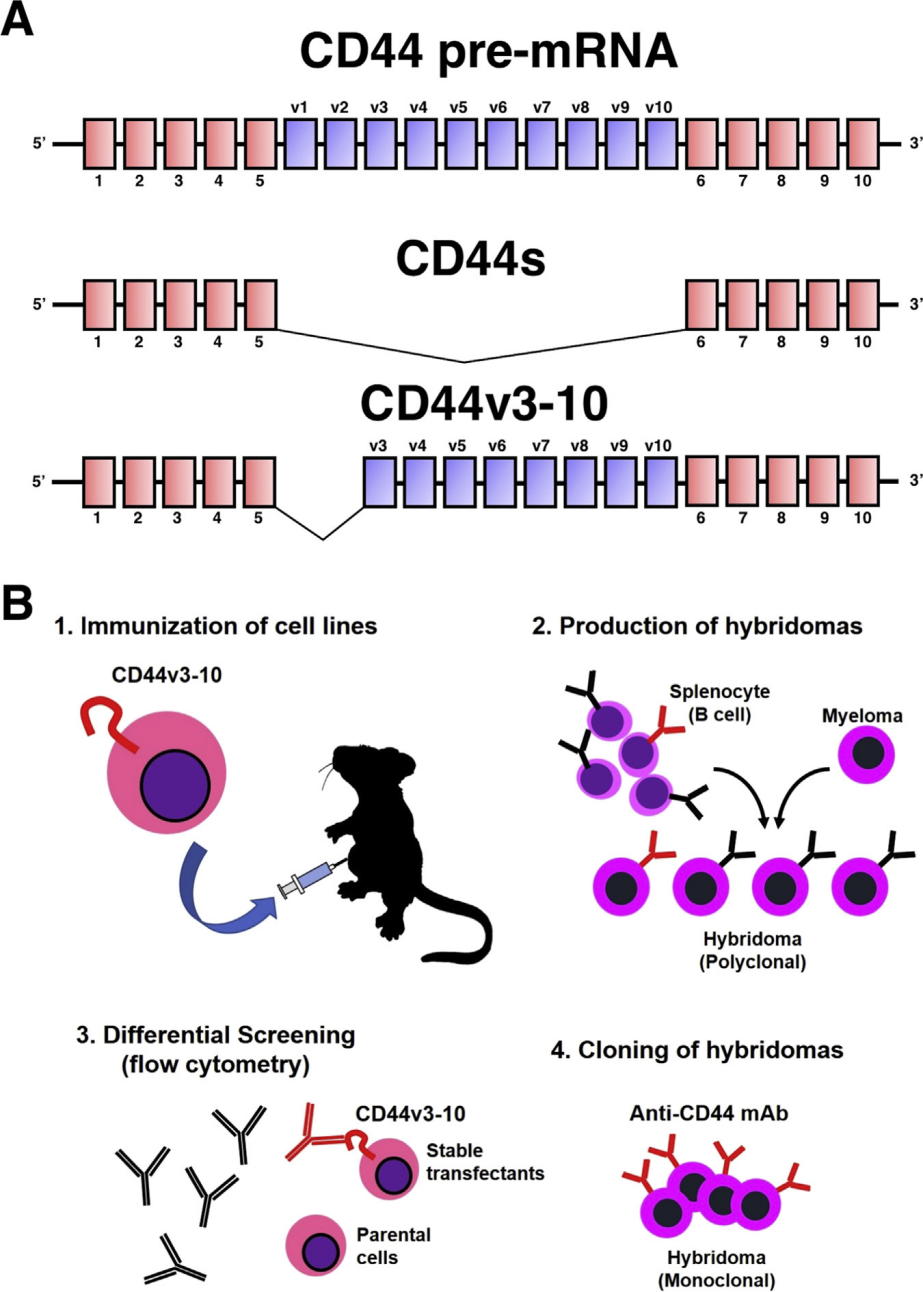
(A) Schematic illustration of CD44 standard (CD44s) and variants (CD44v3-10). (B) Procedure of Cell-Based Immunization and Screening (CBIS) method.

We immunized two mice with CHO/CD44v3-10 cells (Fig. 1A), and performed flow cytometric screening. We previously named this cell-based strategy as the CBIS method, as shown in Fig. 1B. Culture supernatants of 1008 wells were mixed with CHO/CD44v3-10 and CHO-K1 cells, and 15 wells (15/1008; 1.5%) showing a stronger reaction against CHO/CD44v3-10 cells than CHO-K1 cells were ultimately selected. After limiting dilution, 7 clones were established, all of which were classified as IgG_1_ subclass. Clone C_44_Mab-5 (IgG_1_, kappa) reacted with oral cancer tissues in further immunohistochemical analyses (data not shown).

### 3.2 Flow cytometric analysis against oral cancer cell lines

**Fig. 2:**
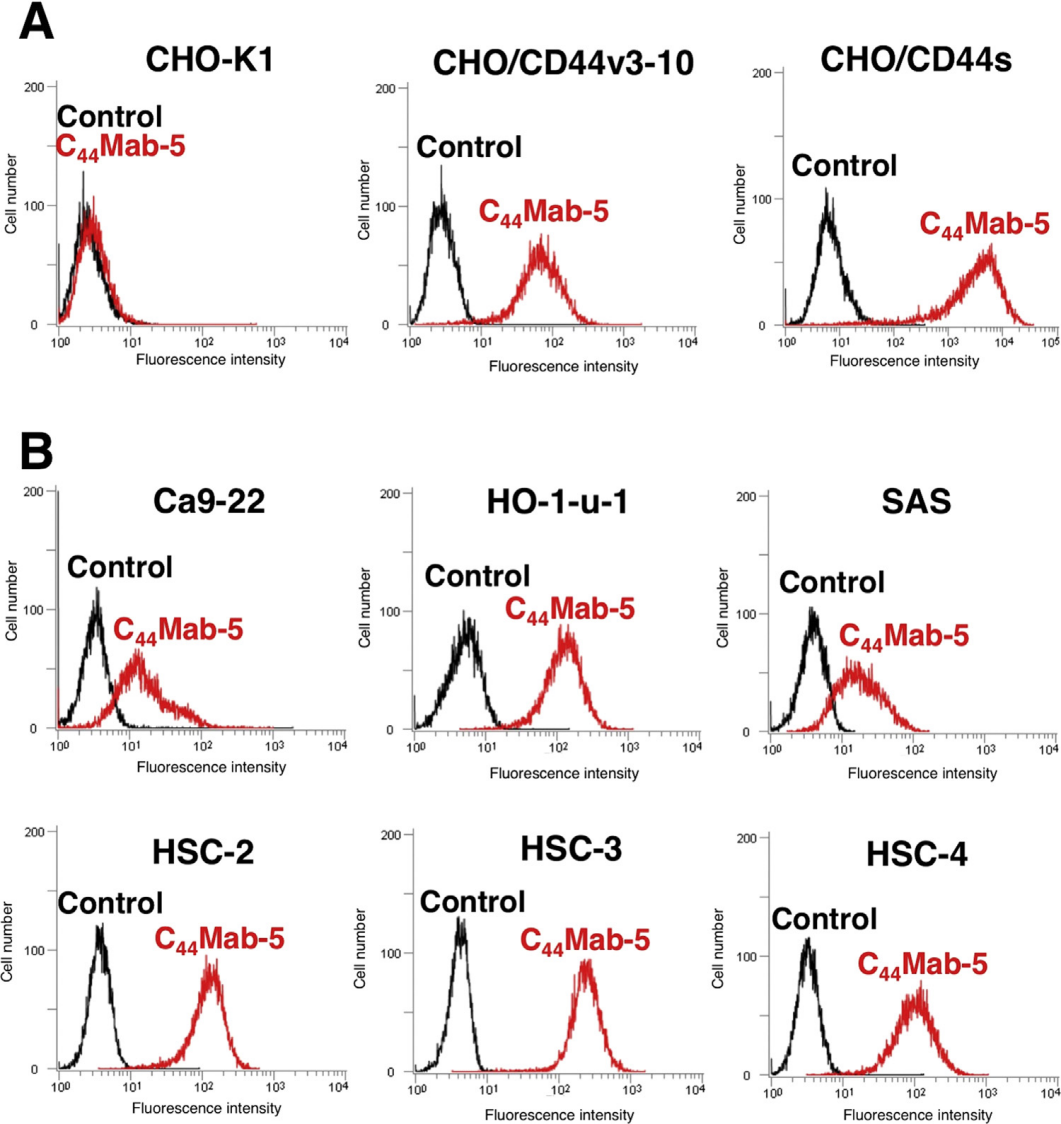
**Flow cytometry of C_44_Mab-5 against oral cancer cell lines**. Cells were treated with 10 μg/mL of C_44_Mab-5 (red line), followed by treatment with Alexa Fluor 488-conjugated anti-mouse IgG; black line, negative control.

C_44_Mab-5 reacted not only with CHO/CD44v3-10 but also with CHO/CD44s (Fig. 2A), indicating that C_44_Mab-5 can detect all CD44 isoforms (Fig. 1A). C_44_Mab-5 also recognized endogenous CD44 in oral cancer cell lines, such as Ca9-22, HO-1-u-1, SAS, HSC-2, HSC-3, and HSC-4 (Fig. 2B).

### 3.3 Immunohistochemical analysis against oral cancer tissues

**Fig. 3:**
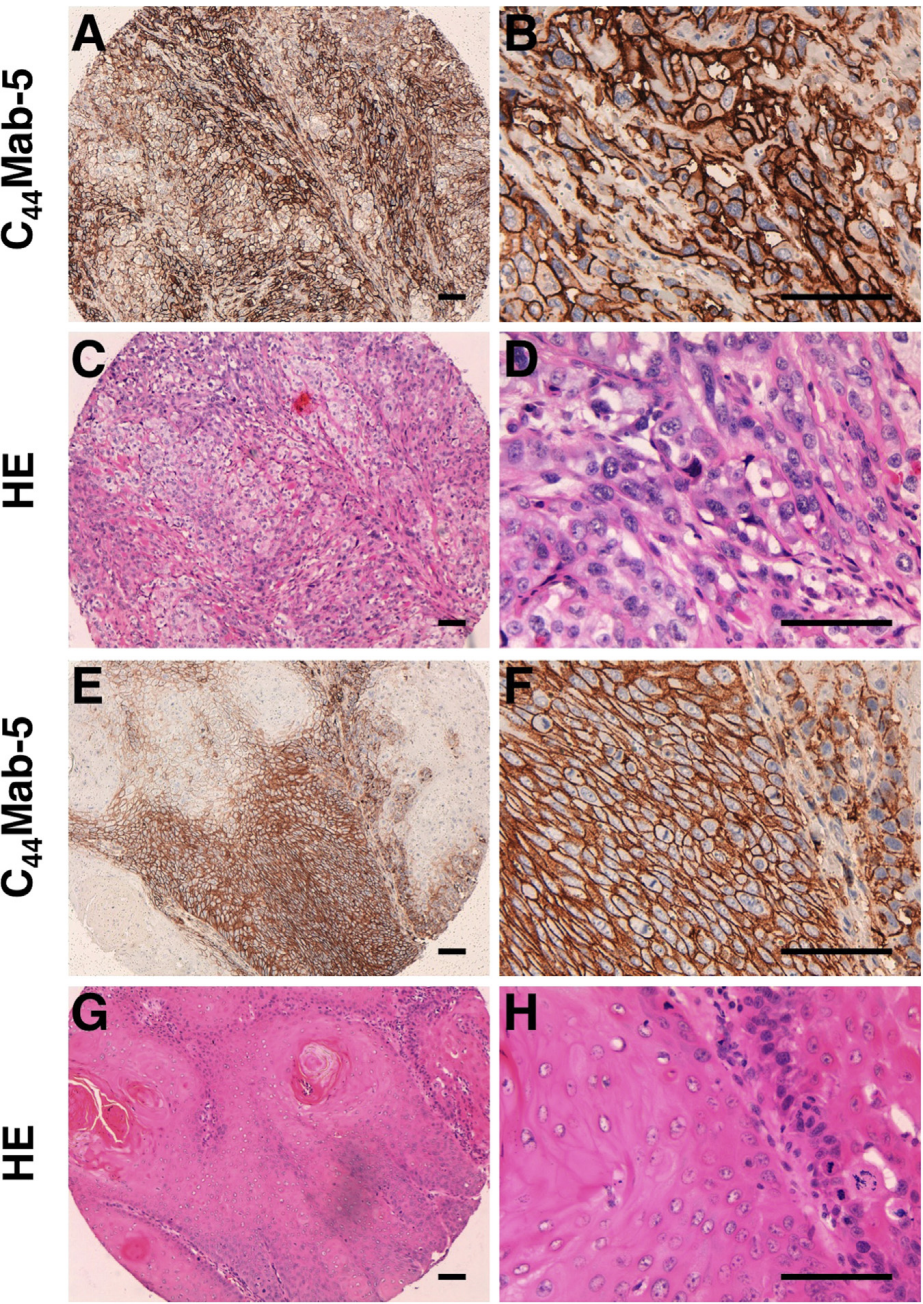
**Immunohistochemical analysis of C**_**44**_**Mab-5 against oral SCCs.** (A, B, E, F) After antigen retrieval, sections were incubated with 1 μg/mL of C_44_Mab-5 followed by treatment with the Envision+ kit. The color was developed using 3,3-diaminobenzidine tetrahydrochloride (DAB), and sections were counterstained with hematoxylin. (C, D, G, H) Hematoxylin & eosin staining (HE). (A-D, case 96; E-H, case 99). Scale bar = 100 µm.

**Fig. S1:**
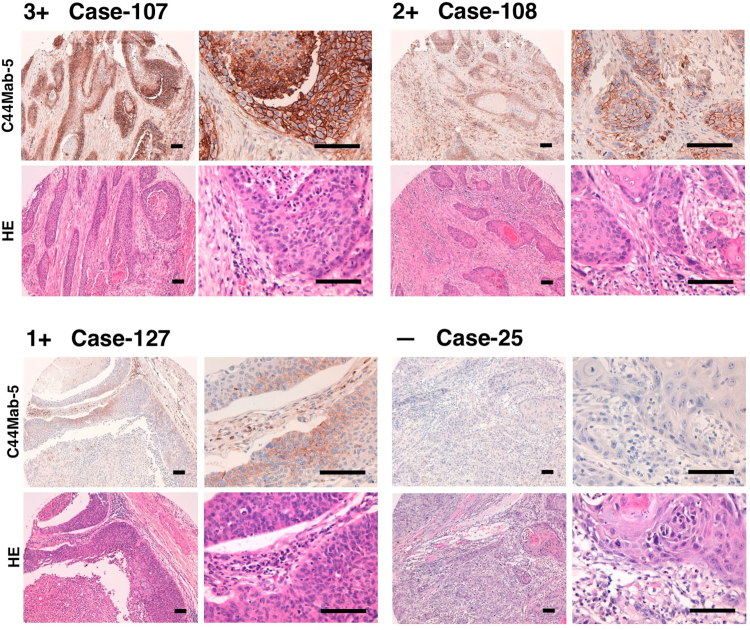
**Immunohistochemical analysis of C**_**44**_**Mab-5 against oral SCCs.** After antigen retrieval, sections were incubated with 1 μg/mL of C_44_Mab-5 followed by treatment with the Envision+ kit. The color was developed using 3,3-diaminobenzidine tetrahydrochloride (DAB), and sections were counterstained with hematoxylin. Hematoxylin & eosin staining (HE) was also performed. Scale bar = 100 µm.

We further investigated the immunohistochemical utility of C_44_Mab-5 in human oral cancers. C_44_Mab-5 stained oral cancer cells in a membrane-staining pattern (Fig. 3), indicating that C_44_Mab-5 may be very useful for immunohistochemical analysis against oral cancers. We then stained oral cancer tissue arrays; typical results (staining level, 3+, 2+, 1+, −) are shown in Supplementary Fig. 1. As shown in Table 1 and Supplementary Table 1, 158/172 (91.9%) of SCCs were stained by C_44_Mab-5, and 80/182 (44.0%) were diagnosed as 3+. Although the samples were limited, adenoid cystic carcinomas (ACCs) and mucoepidermoid carcinomas (MECs) also showed positive staining by C_44_Mab-5.

**Table 1:** Summary of immunostaining using C_44_Mab-5.

Tumor type	No. of cases	C_44_Mab-5 immunostaining				No. of positive cases
		−	1+	2+	3+	
SCC	172	14	20	61	77	158/172 (91.9%)
ACC	7	2	3	1	1	5/7 (71.4%)
MEC	3	0	1	0	2	3/3 (100%)
Total	182	16	24	62	80	166/182 (91.2%)

SCC; squamous cell carcinoma, ACC; adenocystic carcinoma MEC; mucoepidermoid carcinoma.

The intensity of staining was evaluated as −, 1+, 2+, 3+.

In conclusion, we successfully produced C_44_Mab-5, a novel anti-CD44s mAb, using the CBIS method. This method is advantageous because it does not require the membrane protein to be purified in all steps of mAb production. C_44_Mab-5 appears to be promising for the detection of CD44s, as well as many CD44 variants using flow cytometry and immunohistochemical analysis. Future studies investigating the reactivity of C_44_Mab-5 towards other human cancers are warranted.
