# Supplementary material for: Detection of high CD44 expression in oral cancers using the novel monoclonal antibody, C44Mab-5
Source: Biochem Biophys Rep. 2018 Apr 12;14:64–8. doi: 10.1016/j.bbrep.2018.03.007 (PMC5986985; doi:10.1016/j.bbrep.2018.03.007)
Supplement: Supplementary file 2 — Supplementary material [file mmc2.docx]

Supplemental Table 1. Results of C44Mab-5 immunostaining in 182 patients with oral cancers.

| Case  No. | Age | Sex | Organ | Tumor  type | Differentiation | TNM | C44Mab-5 |
| --- | --- | --- | --- | --- | --- | --- | --- |
| 1 | 61 | M | Tongue | SCC | Well | - | 2+ |
| 2 | 57 | F | Tongue | SCC | Well | - | 2+ |
| 3 | 67 | F | Tongue | SCC | Well | - | 3+ |
| 4 | 59 | M | Tongue | SCC | Well | - | 3+ |
| 5 | 47 | F | Tongue | SCC | Well | - | 1+ |
| 6 | 62 | F | Tongue | SCC | - | - | 1+ |
| 7 | 47 | F | Tongue | SCC | Well | - | 2+ |
| 8 | 51 | M | Tongue | SCC | Well | - | - |
| 9 | 62 | M | Tongue | SCC | Well | - | 1+ |
| 10 | 53 | F | Tongue | SCC | Well | - | 2+ |
| 11 | 50 | M | Tongue | SCC | Well | - | 2+ |
| 12 | 76 | M | Tongue | SCC | Well | - | 2+ |
| 13 | 55 | M | Tongue | SCC | Well | - | 2+ |
| 14 | 57 | F | Tongue | SCC | Well | - | 1+ |
| 15 | 61 | M | Tongue | SCC | Well | - | 1+ |
| 16 | 50 | F | Tongue | SCC | Well | - | 2+ |
| 17 | 54 | M | Tongue | SCC | Well | - | 2+ |
| 18 | 62 | M | Tongue | SCC | Well | - | 1+ |
| 19 | 55 | F | Tongue | SCC | Well | - | 2+ |
| 20 | 63 | F | Tongue | SCC | Well | - | 1+ |
| 21 | 56 | M | Tongue | SCC | Well | - | 2+ |
| 22 | 45 | F | Tongue | SCC | Well | - | 2+ |
| 23 | 50 | M | Tongue | SCC | Well | - | 1+ |
| 24 | 46 | F | Tongue | SCC | Poorly | - | - |
| 25 | 48 | F | Tongue | SCC | Moderately | - | - |
| 26 | 67 | F | Tongue | SCC | - | - | 3+ |
| 27 | 64 | M | Tongue | SCC | Poorly | - | 2+ |
| 28 | 50 | F | Tongue | SCC | Moderately | - | 2+ |
| 29 | 63 | M | Tongue | SCC | Moderately | - | 2+ |
| 30 | 46 | F | Tongue | SCC | Well | - | 3+ |
| 31 | 35 | F | Tongue | SCC | Moderately | - | 1+ |
| 32 | 55 | M | Tongue | SCC | Poorly | - | - |
| 33 | 49 | M | Tongue | SCC | Poorly | - | 2+ |
| 34 | 61 | M | Tongue | SCC | Moderately | - | 1+ |
| 35 | 53 | M | Tongue | SCC | Moderately | - | 1+ |
| 36 | 51 | F | Tongue | SCC | Poorly | - | - |
| 37 | 73 | M | Tongue | SCC | Poorly | - | 1+ |
| 38 | 61 | M | Tongue | SCC | Poorly | - | 2+ |
| 39 | 78 | M | Tongue | SCC | Well | T2N0M0 | 1+ |
| 40 | 51 | F | Tongue | SCC | Well | T4N0M0 | 1+ |
| 41 | 75 | F | Tongue | SCC | Well | T2N0M0 | - |
| 42 | 69 | M | Tongue | SCC | Well | T3N0M0 | 3+ |
| 43 | 56 | F | Tongue | SCC | Well | T2N0M0 | 2+ |
| 44 | 35 | F | Tongue | SCC | Well | T2N0M0 | 2+ |
| 45 | 39 | F | Tongue | SCC | Well | T1N0M0 | 2+ |
| 46 | 77 | F | Tongue | SCC | Moderately | T1N0M0 | 2+ |
| 47 | 41 | F | Tongue | SCC | Well | T2N0M0 | 1+ |
| 48 | 53 | M | Tongue | SCC | Well | T2N0M0 | - |
| 49 | 50 | M | Tongue | SCC | Well | T3N0M0 | 3+ |
| 50 | 36 | F | Tongue | SCC | Moderately | T1N0M0 | 2+ |
| 51 | 58 | M | Tongue | SCC | Well | T1N0M0 | 2+ |
| 52 | 63 | F | Tongue | SCC | Well | T1N0M0 | 2+ |
| 53 | 55 | F | Tongue | SCC | Moderately | T2N0M0 | 1+ |
| 54 | 76 | M | Tongue | SCC | Well | T1N0M0 | 1+ |
| 55 | 44 | M | Tongue | SCC | Well | T2N1M0 | 3+ |
| 56 | 53 | F | Tongue | SCC | Well | T1N0M0 | 2+ |
| 57 | 67 | F | Tongue | SCC | Well | T2N0M0 | 3+ |
| 58 | 60 | M | Tongue | SCC | - | T1N0M0 | 3+ |
| 59 | 55 | M | Tongue | SCC | Well | T1N0M0 | 1+ |
| 60 | 61 | M | Tongue | SCC | Well | T1N0M0 | 3+ |
| 61 | 55 | M | Tongue | SCC | Well | T1N0M0 | 2+ |
| 62 | 59 | M | Tongue | SCC | Well | T2N0M0 | 3+ |
| 63 | 46 | F | Tongue | SCC | Well | T2N0M0 | 3+ |
| 64 | 45 | F | Tongue | SCC | Well | T2N0M0 | 3+ |
| 65 | 61 | M | Tongue | SCC | Well | T2N0M0 | 3+ |
| 66 | 48 | F | Tongue | SCC | Well | T2N0M0 | 1+ |
| 67 | 52 | F | Tongue | SCC | Well | T1N0M0 | 3+ |
| 68 | 64 | M | Tongue | SCC | Well | T2N0M0 | 2+ |
| 69 | 46 | F | Tongue | SCC | Well | T2N0M0 | - |
| 70 | 48 | F | Tongue | SCC | Well | T1N0M0 | - |
| 71 | 80 | M | Tongue | SCC | Well | T1N0M0 | 2+ |
| 72 | 49 | M | Tongue | SCC | - | T1N0M0 | - |
| 73 | 60 | M | Tongue | SCC | Well | T2N0M0 | 2+ |
| 74 | 57 | M | Tongue | SCC | Well | T1N0M0 | 3+ |
| 75 | 45 | M | Tongue | SCC | Well | T2N0M0 | 3+ |
| 76 | 47 | F | Tongue | SCC | Well | T2N0M0 | 2+ |
| 77 | 37 | M | Tongue | SCC | Well | T2N1M0 | - |
| 78 | 60 | M | Tongue | SCC | Moderately | T2N0M0 | 2+ |
| 79 | 40 | F | Tongue | SCC | Poorly | T2N0M0 | - |
| 80 | 49 | M | Tongue | SCC | Moderately | T1N0M0 | 3+ |
| 81 | 50 | M | Tongue | SCC | Poorly | T2N0M0 | 2+ |
| 82 | 60 | M | Tongue | SCC | Poorly | T1N0M0 | 2+ |
| 83 | 56 | F | Tongue | SCC | Poorly | T2N0M0 | - |
| 84 | 77 | M | Tongue | SCC | Poorly | T2N0M0 | 2+ |
| 85 | 56 | M | Tongue | SCC | Moderately | T2N1M0 | - |
| 86 | 35 | M | Tongue | SCC | Well | T2N0M0 | 2+ |
| 87 | 52 | F | Soft palate | MEC | - | - | 3+ |
| 88 | 58 | M | Tongue | SCC | Moderately | T2N1M0 | 2+ |
| 89 | 61 | M | Tongue | SCC | Well | T2N0M0 | 2+ |
| 90 | 45 | M | Tongue | SCC | Well | T2N0M0 | 2+ |
| 91 | 72 | M | Tongue | SCC | Poorly | T2N0M0 | 3+ |
| 92 | 46 | F | Gums | SCC | Well | T2N2M0 | 3+ |
| 93 | 61 | M | Tongue | SCC | Moderately | T2N1M0 | 2+ |
| 94 | 21 | F | Palate | MEC | Well | - | 3+ |
| 95 | 54 | M | Tongue | SCC | Poorly | T2N1M0 | 3+ |
| 96 | 58 | M | Tongue | SCC | Moderately | T2N1M0 | 3+ |
| 97 | 44 | F | Palate | MEC | - | T2N1M0 | 1+ |
| 98 | 64 | F | Tongue | SCC | Well | T2N0M0 | 3+ |
| 99 | 56 | M | Gums | SCC | Moderately | T3N0M0 | 3+ |
| 100 | 54 | M | Gums | SCC | Moderately | T1N0M0 | 2+ |
| 101 | 59 | F | Tongue | SCC | Moderately | T2N0M0 | 3+ |
| 102 | 57 | M | Tongue | SCC | Well | T1N2M0 | 3+ |
| 103 | 5 | M | Tongue | SCC | Moderately | T2N2M0 | 3+ |
| 104 | 43 | M | Tongue | SCC | Poorly | T3N0M0 | 3+ |
| 105 | 61 | M | Tongue | SCC | Well | T2N1M0 | 3+ |
| 106 | 64 | M | Mouth floor | SCC | Well | - | 3+ |
| 107 | 57 | M | Tongue | SCC | Moderately | T1N0M0 | 3+ |
| 108 | 67 | M | Tongue | SCC | Well | T4N1M0 | 2+ |
| 109 | 50 | M | Tongue | SCC | Moderately | T1N1M0 | 2+ |
| 110 | 65 | M | Tongue | SCC | Well | T2N2M0 | 2+ |
| 111 | 57 | F | Soft palate | ACC | - | T1N0M0 | 2+ |
| 112 | 71 | M | Tongue | SCC | Well | T3N0M0 | 2+ |
| 113 | 57 | M | Tongue | SCC | Poorly | T3N0M0 | 2+ |
| 114 | 54 | M | Soft palate | SCC | Moderately | T3N0M0 | 3+ |
| 115 | 53 | M | Tongue | SCC | Well | T3N1M0 | 3+ |
| 116 | 59 | M | Gums | SCC | Well | T1N0M0 | 3+ |
| 117 | 58 | F | Gums | SCC | Well | T2N0M0 | 2+ |
| 118 | 47 | F | Palate | ACC | - | T3N0M0 | 1+ |
| 119 | 74 | M | Tongue | ACC | - | T2N0M0 | 1+ |
| 120 | 53 | M | Tongue | SCC | Well | T1N0M0 | 3+ |
| 121 | 57 | F | Tongue | SCC | Well | T2N1M0 | 2+ |
| 122 | 36 | M | Tongue | SCC | Well | T1N0M0 | 3+ |
| 123 | 44 | M | Buccal mucosa | SCC | Moderately | T3N0M0 | 2+ |
| 124 | 77 | M | Tongue | SCC | Well | T3N0M0 | 3+ |
| 125 | 43 | F | Tongue | SCC | Poorly | T3N0M0 | 2+ |
| 126 | 65 | M | Tongue | ACC | - | T2N0M0 | 3+ |
| 127 | 80 | M | Gums | SCC | Moderately | T1N1M0 | 1+ |
| 128 | 69 | M | Left mandible | SCC | Well | T2N1M0 | 2+ |
| 129 | 76 | F | Gums | SCC | - | T2N1M0 | 3+ |
| 130 | 76 | F | Tongue | SCC | Well | T3N0M0 | 2+ |
| 131 | 65 | F | Tongue | SCC | Well | T4N0M0 | 3+ |
| 132 | 42 | M | Mouth floor | SCC | Well | T1N0M0 | 2+ |
| 133 | 60 | F | Tongue | SCC | Well | T1N0M0 | 2+ |
| 134 | 58 | M | Tongue | SCC | Moderately | T3N1M0 | 3+ |
| 135 | 52 | M | Tongue | SCC | Well | T1N0M0 | 3+ |
| 136 | 60 | F | Tongue | SCC | Well | T2N0M0 | 3+ |
| 137 | 43 | F | Tongue | SCC | Well | T3N0M0 | 2+ |
| 138 | 40 | F | Tongue | SCC | Well | T2N0M0 | 2+ |
| 139 | 49 | M | Tongue | SCC | Well | T1N0M0 | 2+ |
| 140 | 69 | M | Pharynx | SCC | Well | T4N2M0 | 3+ |
| 141 | 54 | M | Tongue | SCC | Moderately | T2N0M0 | 2+ |
| 142 | 63 | F | Gums | SCC | Well | T2N1M0 | 2+ |
| 143 | 51 | F | Soft palate | ACC | - | T1N0M0 | - |
| 144 | 69 | F | Gums | SCC | Well | T2N0M0 | 3+ |
| 145 | 48 | M | Palate | ACC | - | T2N0M0 | - |
| 146 | 49 | M | Tongue | ACC | - | T2N1M0 | 1+ |
| 147 | 55 | M | Mouth floor | SCC | Well | T2N1M1 | 3+ |
| 148 | 63 | F | Tongue | SCC | Well | T2N0M0 | 3+ |
| 149 | 47 | M | Tongue | SCC | Well | T2N1M0 | 3+ |
| 150 | 49 | M | Tongue | SCC | Moderately | T2N0M0 | 3+ |
| 151 | 61 | M | Tongue | SCC | Well | T1N0M0 | 3+ |
| 152 | 71 | M | Palate | SCC | Moderately | T3N1M0 | 3+ |
| 153 | 47 | M | Tongue | SCC | Well | T2N1M0 | 3+ |
| 154 | 60 | M | Mouth floor | SCC | Poorly | T2N0M0 | 3+ |
| 155 | 58 | M | Tongue | SCC | Moderately | T3N0M0 | 3+ |
| 156 | 49 | F | Tongue | SCC | Poorly | T3N0M0 | 2+ |
| 157 | 67 | M | Tongue | SCC | Well | T2N0M0 | 2+ |
| 158 | 64 | M | Tongue | SCC | Moderately | T2N0M0 | 3+ |
| 159 | 55 | M | Tongue | SCC | Moderately | T2N0M0 | 3+ |
| 160 | 51 | M | Tongue | SCC | Well | T1N0M0 | 3+ |
| 161 | 73 | M | Tongue | SCC | Well | T1N0M0 | 3+ |
| 162 | 34 | F | Tongue | SCC | Well | T1N0M0 | 3+ |
| 163 | 29 | F | Tongue | SCC | Well | T1N0M0 | 3+ |
| 164 | 43 | F | Tongue | SCC | Moderately | T2N0M0 | 3+ |
| 165 | 29 | F | Tongue | SCC | Well | T1N0M0 | 3+ |
| 166 | 52 | F | Tongue | SCC | Moderately | T1N0M0 | 3+ |
| 167 | 39 | M | Tongue | SCC | Well | T2N1M0 | 3+ |
| 168 | 49 | M | Tongue | SCC | Well | T2N2bM0 | 3+ |
| 169 | 67 | M | Tongue | SCC | Well | T2N0M0 | 3+ |
| 170 | 69 | M | Tongue | SCC | Well | T1N0M0 | 3+ |
| 171 | 35 | F | Tongue | SCC | Moderately | T2N0M0 | 3+ |
| 172 | 55 | F | Tongue | SCC | Well | T2N0M0 | 3+ |
| 173 | 65 | M | Tongue | SCC | Moderately | T3N2aM0 | 3+ |
| 174 | 75 | M | Tongue | SCC | Well | T2N1M0 | 3+ |
| 175 | 64 | M | Tongue | SCC | Well | T2N1M0 | 3+ |
| 176 | 78 | M | Tongue | SCC | Well | T2N0M0 | 3+ |
| 177 | 50 | F | Tongue | SCC | Well | T2N0M0 | 3+ |
| 178 | 48 | M | Tongue | SCC | Well | T1N0M0 | 3+ |
| 179 | 64 | M | Tongue | SCC | Moderately | T2N0M0 | 3+ |
| 180 | 50 | M | Tonue | SCC | Moderately | T3N1M0 | 2+ |
| 181 | 65 | M | Tongue | SCC | Moderately | T3N0M0 | 3+ |
| 182 | 38 | M | Tongue | SCC | Well | T3N0M0 | 3+ |

SCC; squamous cell carcinoma, ACC; adenocystic carcinoma
MEC; mucoepidermoid carcinoma

The intensity of staining was evaluated as -, 1+, 2+, 3+
